# Dormancy and awakening of cancer cells: the extracellular vesicle-mediated cross-talk between Dr. Jekill and Mr. Hyde

**DOI:** 10.3389/fimmu.2024.1441914

**Published:** 2024-09-05

**Authors:** Concetta D’Antonio, Giovanna L. Liguori

**Affiliations:** Institute of Genetics and Biophysics (IGB) “Adriano Buzzati-Traverso”, National Research Council (CNR) of Italy, Naples, Italy

**Keywords:** extracellular vesicles, cancer cell dormancy, cancer cell awakening, tumor microenvironment, premetastatic niche, metastasis, cancer diagnosis and therapy, EV dormancy signature

## Abstract

Cancer cell dormancy is a reversible process whereby cancer cells enter a quiescent state characterized by cell cycle arrest, inhibition of cell migration and invasion, and increased chemoresistance. Because of its reversibility and resistance to treatment, dormancy is a key process to study, monitor, and interfere with, in order to prevent tumor recurrence and metastasis and improve the prognosis of cancer patients. However, to achieve this goal, further studies are needed to elucidate the mechanisms underlying this complex and dynamic dual process. Here, we review the contribution of extracellular vesicles (EVs) to the regulation of cancer cell dormancy/awakening, focusing on the cross-talk between tumor and non-tumor cells in both the primary tumor and the (pre-)metastatic niche. Although EVs are recognized as key players in tumor progression and metastasis, as well as in tumor diagnostics and therapeutics, their role specifically in dormancy induction/escape is still largely elusive. We report on the most recent and promising results on this topic, focusing on the EV-associated nucleic acids involved. We highlight how EV studies could greatly contribute to the identification of dormancy signaling pathways and a dormancy/early awakening signature for the development of successful diagnostic/prognostic and therapeutic approaches.

## 1 Introduction

In tumorigenesis, two different levels of dormancy have been distinguished so far. The first level, called tumor dormancy, was first proposed by Judah Folkman in the early 1970s and indicates the whole tumor mass maintaining an overall constant size possibly due to a balance between cell proliferation and death (1, 2). The second level, called cancer cell dormancy or quiescence is a process whereby individual tumor cells inside the tumor enter a state of cell cycle arrest (3). Cancer cell entry into a dormant state can occur at any stage of cancer progression, even the ones characterized by fast tumor growth, and in different types of tumors, including the highly aggressive and metastatic cancers (4, 5). In this Review we will explore the dormancy at a cellular level. Besides cell cycle arrest, dormant cancer cells are also characterized by the acquisition of stemness, mesenchymal and chemotolerance features and the activation of the related signaling pathways, involving Notch, Transforming Growth Factor β (TGFβ)as well as Bone Morphogenetic Protein (BMP) receptors, and the Zinc finger E-box-binding homeobox (ZEB2) transcription factor (6–10). Dormant cancer cells resemble, and in some cases, overlap with quiescent/slow proliferating cancer stem cells, thus suggesting that they are different metastable states, rather than separate entities, along the same cell continuum (10). Stemness pathways activated in dormant cells can be also active in quiescent/slow-proliferating cancer stem cells and even in quiescent normal stem cells, such as the long-term hematopoietic stem cells (LT-HSCs). LT-HSCs are a subset of HSCs that remain quiescent to guarantee the existence of an HSC pool and the continuous production of blood cells throughout an individual’s lifetime (11). Interestingly, the hematopoietic cell kinase, normally expressed in the lymphoid and myeloid lineages of hemopoiesis (12), has been found to be enriched in dormant leukemia cells, interfering with the maturation of the FMS-like tyrosine kinase 3 (Flt3) and causing abnormal Flt3 signaling (13).

Dormancy is not associated to gene mutations, but to a specific epigenetic signature. Indeed, it is a reversible process, which means that quiescent cancer cells can awake in every moment, even after many years, and restart proliferating giving rise according to the specific context to the primary tumor, to tumor relapse after excision and or (chemo)therapy, or to formation of metastasis at distant sites (8, 10). In both melanoma and glioblastoma (GB) a restricted and relatively quiescent subset of cancer cells was identified as responsible for sustained long-term tumor growth (14, 15). In addition to these two tumors, dormancy/reawakening balance has been mainly identified in breast and prostate cancer cells, as well as in brain, liver, lung and bone metastatic niche (16–19). The discovery of tumor quiescence revolutionized the concept of tumor progression and metastasis, suggesting that the initial mutations that give rise to cancer cells, as well as the dissemination of cancer cells to distant sites, may precede the onset of tumor and metastasis respectively, even by many years (20–23). The therapy resistance and the reversibility that characterize dormant state make it extremely important to monitor and crucial to regulate with the perspective to contrast tumor initiation, progression, relapse and metastasis.

Tumor microenvironment plays a key role in regulating cancer cell dormancy/awakening at both primary tumor and (pre-)metastatic sites. Premetastatic niche are actively formed by extracellular signals emanating from the primary tumors, which modify the microenvironment at distant sites, rendering it supportive for survival and outgrowth of circulating tumor cells long before their arrival, colonization and engraftment (24, 25). Studies on the pre-metastatic niche highlighted that dormancy is a very specific process, supported by a unique microenvironment specific to each cancer type (19, 23). As an example, the microvascular endothelium is able to induce dormancy in disseminated breast cancer cells, whereas the osteoblastic niche can promote quiescence of prostate cancer and multiple myeloma cells (26–29). In each niche, specific factors are involved in dormancy induction, released by non-tumor cells, such as stromal, endothelial and immune cells, as well as present in the Extracellular Matrix (ECM) (30–33). ECM components such as thrombospondin-1 and osteopontin have been associated to induction of dormancy in disseminated tumor cells (DTCs), respectively in the perivascular and osteoblastic niches (26, 34). Induction of DTC quiescency can be also mediated by soluble factors, including the growth arrest-specific protein 6 (GAS6), Transforming Growth Factor β2 (TGFβ2), bone morphogenetic protein 4 and 7 (BMP4 and 7) (16, 35–37). Soluble and ECM factors cooperate in regulation of quiescence, as shown for the ECM component fibronectin, whose production is dependent from the cancer-secreted TGFβ2 which is able to induce DTC quiescence (38). However, despite the fact that each niche microenvironment uses specific factors to induce quiescence in cancer cells, the downstream intracellular signaling pathways commonly activated are the ERK signaling, C-X-C chemokine receptor type 4 (CXCR4) activated Src-dependent signaling, endoplasmic reticulum stress, VCAM1, Wnt and BMP-dependent signaling (19, 32, 33, 37, 39, 40).

Extracellular vesicles (EVs) are key components of the tumor microenvironment and (pre-)metastatic niche, being produced by both tumor and non-tumor cells and being able to transport active molecules, such as DNAs, RNAs (mRNAs, lncRNAs and miRNAs), proteins, lipids and metabolites to target cells (both tumoral and non), thereby influencing their state (41, 42). EVs can be closely associated with tissues or the ECM, and are referred to as tissue-derived EVs (Ti-EVs) or matrix-bound vesicles (MBVs) (87, 88). Finally, EVs can be also transported in body fluids reaching target sites at very long distance from the producing cells, with relevant diagnostic and therapeutic implications (41, 42). Based on their biogenesis, two main types of vesicles have been defined: the exosomes, which are formed in multivesicular bodies inside cells and then secreted outside, and the microvesicles or ectosomes, which originate from cell membrane budding (89). During tumor development, EVs have been shown to play a key role in many processes related to cell communication, being involved in the induction of stemness, epithelial-mesenchymal transition (EMT), proliferation, migration, chemoresistance and metastasis formation (42, 43, 90). However, the specific contribution of EVs to the induction and regulation of tumor quiescence is still in its infancy. Therefore, our review article aims to explore the impact of EVs on the dormancy/awakening balance by summarizing more relevant and recent studies on this topic, and discuss the potentiality in using EVs for theranostic dormancy/awakening targeting approaches.

## 2 The contribution of extracellular vesicles to dormancy/awakening balance

**Figure 1:**
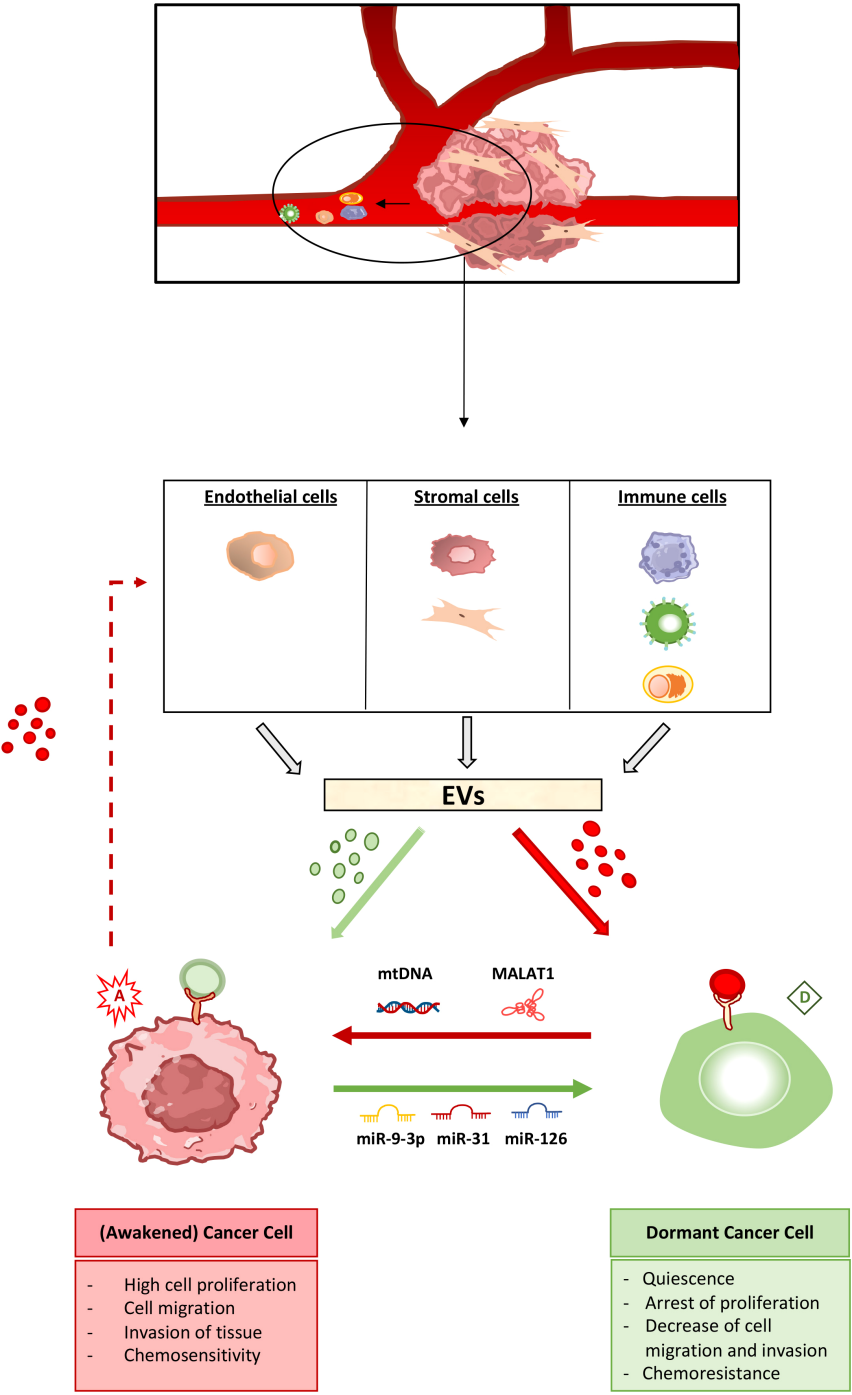
The extracellular vesicle-mediated cross-talk in the regulation of cancer cell dormancy and awakening. Cancer cell dormancy is a reversible process. Non-tumor cells in the tumor microenvironment as well as at the (pre-)metastatic site, including endothelial, stromal and immune cells, produce extracellular vesicles (EVs) capable of inducing cancer cell dormant (green) as well as awakening (red) state. Pro-dormancy EVs (shown in green) target cancer cells and induce the activation of intracellular pathways leading to arrest of cell proliferation, migration and invasion together to increased chemoresistance. Pro-awakening vesicles (shown in red) act on dormant tumor cells, inducing the escape from quiescence, with increased cell proliferation and migration, the ability to invade other tissues as well as acquired chemosensitivity. The EV cargo capable of shifting the balance between dormant and awakening state is also highlighted. miR-9-3p, miR-31 and miR-126 are among the microRNAs able of promoting dormancy of cancer cells, whereas mitochondrial DNA (mtDNA) and the MALAT1 long non-coding RNA induce cancer cell awakening. Cancer cells, in turn, produce EVs that act on non-tumor cells stimulating the production and release of primed EVs, that can both induce and reverse the dormancy process.

A complete characterization of dormant cancer cells has not yet been achieved, so it is challenging to clearly assess the contribution of external signals, including EVs, to dormancy induction. Here we collect evidence on the ability of EVs released by non-tumoral cells in the tumor or (pre-)metastatic niche, such as stromal, immune and endothelial cells, to promote one or more features related to dormancy. The ability of the tumor microenvironment cells to regulate dormancy/awakening balance is illustrated in **Figure 1**.

Several *in vitro* studies have addressed the ability of stromal EV cargo to decrease tumorigenic features, such as proliferation, migration, and invasion, and induce a quiescent-like and highly chemoresistant state in cancer cells. Among the different types, metastatic breast cancer cells (BCCs) have been extensively studied, due to the clinical evidence of very late relapses. EV released by stromal cells (fibroblasts and cancer associated fibroblasts or CAFs) have been shown to target BCCs, causing the increase of chemoresistance through activation of Stat1 and Notch3 signaling pathways (44). Similarly, EVs released by bone marrow-derived mesenchymal stem cells (BM-MSCs) are uptaken by BCCs, causing reduction of cell proliferation, migration and invasion, enhanced adhesion, increased chemoresistance and the decrease of stem cell-like markers on the cell membrane (45, 46). In most cases the effect is due to EV transfer of specific miRNAs between stromal and cancer cells; the most relevant miRNAs identified have been summarized in **Table 1**. BM-MSC derived EV miR-23b downregulates the expression of the gene encoding the myristoylated alanine-rich C-kinase substrate (MARCKS) protein, which in turn promotes cell cycling and motility (45). Other stromal EV-associated miRNAs, miR-31 and 205, suppress the metastatic potential of BCCs by repressing the gene encoding Ubiquitin Conjugating Enzyme E2 N (UBE2N/Ubc13), that is involved in cell proliferation, migration and invasion ( (47).

**Table 1:** EV-RNAs released from non-tumor cells and involved in cancer cell dormancy/awakening.

EV cargo	EV Source	Target Cells	Induced Effect	Signaling Pathway	References
miR-23b	BM-MSCs	bone metastatic BCCs	Inhibition of cell invasion; increase of chemoresistance; decrease of stemness markers	MARCKS downregulation	45
miR-31 and -205	MSCs	bone metastatic BCCs	Inhibition of metastatic potential	UBE2N/Ubc13 downregulation	47
miR-127	BM-MSCs primed with BCCs	BCCs	Decreased proliferation; induction of quiescence	Targeting of C-X-C Motif Chemokine Ligand 12 (CXCL12)	91
miR-197	BM-MSCs primed with BCCs	BCCs	Decreased proliferation; induction of quiescence	CXCL12 targeting	91
miR-222/223	BM-MSCs primed with BCCs	BCCs	Induction of cycling quiescence and chemoresistance	CXCL12 targeting	48, 91
miR-9-3p	BM-MSCs	bladder cancer cells	Reduction of cell viability, migration and invasion; induction of apoptosis	Endothelial ell-specific molecule 1 (ESM1) downregulation	49
mir-300	BM-MSCs	Leukemia stem cells	Induction of growth arrest, apoptosis and drug resistance	Protein phosphatase 2A (PP2A) activation	50
miR-126	BM endothelial cells	Leukemia stem cells	Induction of quiescence and persistence, poor prognosis	Unknown	51
MALAT1	Endothelial cells	Metastatic BCCs	Suppression of immunogenic cell death	Activation of Wnt pathway and induction of Serpin protease inhibitors	52, 53

Further studies reported that exposure to BCCs does influence the cargo composition of EVs released by BM-MSCs and can be involved itself in the induction of cycling quiescence and early cancer dormancy (48, 54). A stepwise dormancy process was proposed in which early naive EVs released from BM-MSCs began the BCC transition into cycling quiescence and their reorganization into distinct cell subsets, whereas BCC-primed MSC-derived EVs target a subset of cancer cells and complete the process towards dedifferentiation and acquisition of stemness properties (54). Specific miRNAs (miR-127, miR-197, miR-222/223) found in primed MSC-EVs have been associated with reduced proliferation, and induction of cancer cell dormancy and chemoresistance (48). Studies using liver microphysiological system as a model to recapitulate the early metastatic events also highlighted a timely controlled cross-talk between the hepatic niche and BCCs enabling first liver seeding and then reduced tumor outgrowth and cancer cell entry into dormancy at the metastatic site. Hepatic EVs were able to reduce cancer cell proliferation and invasion and concomitantly revert the EMT, with induction of epithelial markers such as E-cadherin and Zonula Occludens-1 (ZO-1) and the acquisition of an epithelial-like morphology (55). Bone marrow-mesenchymal EVs have been shown to target not only BCCs, but also other types of cancer cells, both solid (bladder cancer) and liquid (chronic myelogenous leukemia), inducing cycling quiescence and increased drug resistance. This activity is mediated by EV miRNAs transfer, in particular miR-9-3p for bladder cancer and miR-300 for leukemia (49, 50).

Noteworthily, MSCs can also release awakening signals able to revert the quiescence process (**Figure 1**). Several studies point to the ability of tumor derived EVs (TDEVs) to condition MSCs and stromal cells to release in the microenvironment pro-tumorigenic signals, such as interleukins (ILs) 6 and 8, vascular endothelial growth factor (VEGF) and monocyte-chemotactic protein-1 (MCP-1), to stimulate angiogenesis and immune suppression, and then supporting cancer cell proliferation and survival (23, 42). EV packaging and transfer of mitochondrial DNA from CAFs to metastatic BCCs is able to induce exit from dormancy through estrogen receptor-independent oxidative phosphorylation (OXPHOS) (56). Interestingly, teratocarcinoma-derived EVs have been shown to inhibit migration of GB cells *in vitro* (57), suggesting that TDEVs could also directly target cancer cells contributing to the induction of a dormant-like state.

Very few data are available on the implication of non-stromal (endothelial and immune) EV cargo in the induction of cancer cell quiescence. Bone marrow endothelial EVs have been shown to transfer miR-126 to leukemia cells, thus supporting quiescence, as well as prolonged risk of cancer relapse and worst prognosis (51). Endothelial EVs seem, instead, more involved in cancer cell awakening. In most cases, in fact, endothelial cells in the tumor niche are conditioned by TDEVs, to release EVs carrying pro-tumorigenic factors, which enhance in turn cancer cell proliferation, migration and invasion (23, 42). In mouse models of tumorigenesis, endothelial EVs were shown to target glioma or metastatic breast cancer, enhancing cancer cell self-renewal and proliferation (52, 58). Very recently, endothelial EVs have been shown to protect tumor dormant cells by T cell mediated immunosuppression, by inducing their reactivation or awakening through transfer of the lncRNA MALAT1. MALAT1 causes activation of Wnt pathway and induction of Serpin protease inhibitors, thus suppressing immunogenic cell death in the incipient metastatic cells (52, 53).

Similarly, only few studies pointed to an implication of immune EV cargo in dormancy induction. As an example, EVs released from pro-tumorigenic M2, but interestingly not anti-tumoral M1, macrophages were able to induce cycling quiescence and drug resistance in BCCs (59). Instead, preceding metastatic outgrowth, TDEVs can target immune cells and induce an immunotolerant microenvironment in which dormant cells can awaken and start proliferating, by blocking myeloid and lymphoid differentiation, promoting pro-tumorigenic M2 macrophage polarization, and/or inducing the expansion of immunosuppressive cells like Treg lymphocytes (60, 61).

Finally, we would highlight that EVs might promote cancer cell dormancy/awakening also indirectly, through complex regulatory networks involving other EV-mediated processes such as autophagy, ECM remodeling, inflammation or hypoxia stress, that are ones of the main triggers of cancer cell quiescence (62). For instance, dormant cancer cells can survive in hypoxic and nutrient deficient tumor microenvironments through autophagic processes (63), as well as they are able to reorganize fibronectin in the ECM to maintain dormancy in breast cancer cell lines (38).

## 3 Diagnostic and therapeutic EV significance to detect/regulate cancer dormancy

EV-associated molecules, including proteins, lipids and nucleic acids, can serve as reliable biomarkers for early-stage cancer detection and classification (41, 42, 64). Therefore, defining EV signatures related to tumor dormancy or early awakening might be crucial to monitor cancer progression, including the risk of late relapses and metastasis. In particular, the possibility of amplifying and coupling miRNAs with high-throughput multiplexed RNA profiling, make miRNAs extremely interesting for theranostic purposes. EV-associated miRNAs listed above and in **Table 1** might be interesting markers to follow in circulating EVs, being their increase in tumor and (pre-)metastatic microenvironment, and body fluids potentially associated to cancer cell dormancy/awakening balance. Moreover, different TDEV-miRNAs, including miR-19a, miR-21, miR-141 and miR-375, have been found associated to cancer relapse and metastasis, both *in vitro* and *in vivo*, thus being potential candidate biomarkers to follow (23, 65–67). However, the low number of dormant cells is an obvious limitation that may affect the sensitivity of liquid biopsy. Tissue and matrix bound vesicles could also be useful, as they may better reflect the pathological state of the primary tumor or (pre-)metastatic niche compared to biofluid EVs. Unfortunately, their isolation is less immediate and more invasive, requiring tissue or matrix isolation and dissociation by enzymatic digestion. However, the study of Ti-EVs and MBVs could help to assess a dormant/pro-awakening EV signature, which could then be investigated by EV analysis via liquid biopsy.

The best therapeutic options to target dormancy and therefore avoid tumor relapse or metastasis are still controversial. The different approaches can be subdivided into four treatment strategies, in some cases based on divergent principles, here summarized: (i) induction of maintenance of cancer cells in a quiescent state throughout the life of the patient, also defined as the “sleeping strategy”, or at the opposite (ii) controlled reactivation of dormant cells to overcome drug tolerance, the so called “awakening strategy”, (iii) inhibition of dormancy, and (iv) elimination of dormant cancer cells from patient’s body (8, 32). The maintenance of dormancy or sleeping strategy is probably the most logical one. The strategy involves the repression of the proliferation signaling pathways (mediated by β1-integrin, uPAR, ERK and Src kinases), the induction of intrinsic dormancy factors, such as the kinases DYRK1A and p38 MAPK, or extrinsic signals produced by the pre-metastatic dormant niche, including GAS6, TGF-β2, BMP4 and 7 and Nuclear factor erythroid 2-related factor 2 (NRF2), which is currently being evaluated in a clinical trial for the therapy of head and neck squamous cell carcinomas (NCT03572387) (8). However, and not secondarily, the sleeping strategy has the great disadvantage of requiring prolonged treatment for the whole life of the patient.

As the opposite, both the awakening approach and the inhibition of dormancy would involve the targeting of dormancy-promoting factors released in the microenvironment (e.g., using neutralizing antibodies against osteopontin) (34) or by inhibiting specific kinases able to induce cell cycle arrest and quiescence (68, 69). The use of these molecules strongly enhances the effect of drugs targeting the proliferative cells, and could potentially being used for combination therapy (8). Last, but not least, the elimination of dormant cancer cells is probably the most translational strategy and can be achieved through administration of different substances such as antibiotics (mithramycin), tyrosine kinase inhibitors (linsitinib), or phosphorylation inhibitors (oligomycin) (8). The ideal therapy should be able to efficiently target dormant cancer cells, while sparing the patient’s quiescent normal stem cells, such as LT-HSCs. Therefore, the identification of differentially expressed molecules and differentially activated pathways between quiescent cancer and normal stem cells is fundamental for the development of safe and efficient targeting approaches.

Finally, the strong limitation of all the approaches described is that in case of efficiency less than 100%, the cells that escape the treatment could show a more malignant phenotype, worsening the patient prognosis.

EV exploitation might be extremely useful for developing new suitable anti-cancer therapeutic approaches with increased efficacy and efficiency (43, 70–72). First of all, EV cargo associated with dormancy/awakening induction (**Table 1**) could be promising targets to be blocked. As an example, *in vivo* targeting of the EV-miR-222/223 associated to BCC dormancy was able to reverse the quiescence process in BCCs, increasing their chemosensitivity and increasing mouse model survival (48). Second, EVs could be extremely useful as biological nanocarriers to deliver anti-dormancy or anti-proliferative drugs, depending on the strategy chosen, as well as to target specific dormancy/awakening factors within cancer dormant cells. EVs derived from different sources are suitable natural drug-delivery vectors, due to their high biocompatibility and safety, ability to cross the physiological boundaries and high immunotolerance (73–77). EVs might be instrumental for all the types of strategies, through engineering with the molecule to deliver, prolonging the half-life and even the efficiency of the carried molecules, respect to the soluble ones. Moreover, membrane functionalization and eventual biomimetic designing (78, 79) might help in recognizing and targeting specific cancer dormant cells through specific cell marker interactions.

## 4 Discussion

As shown in the **Figure 1**, duality strongly characterizes the dormancy induction process: (i) cancer cells can exist both in an active (or awakened) and a dormant state, (ii) the switching from one state to the other is reversible, (iii) the non-tumor cells in the primary tumor or (pre-)metastatic niche can release both pro-dormancy and pro-awakening EVs, and (iv) cancer cells can condition non-tumor cells of the microenvironment to release both type of EV-associated signals.

All these features made us think of the dual character par excellence, namely Dr. Jekill and Mr. Hyde. The transformation of Dr. Jekill in Mr. Hyde is reversible, induced by opposing extrinsic factors (the potion and its antidote). At this metaphoric level, EVs, together with other extrinsic factors, such as soluble or ECM-bound molecules, would function as potion as well as antidote to shift the balance between dormant (Dr. Jekill) and awakening (Mr. Hyde) cells, as highlighted in **Figure 1**. Just as Dr. Jekyll had a hidden malignant nature that fully manifested itself when he became Mr. Hyde, so dormant cancer cells retain the malignant potential to resume proliferation, upon awakening, leading to tumor recurrence or metastasis to distant sites. The high persistence and drug resistance that characterizes dormancy, together with the unpredictable intrinsic risk of awakening, leading to tumor relapse and/or metastasis, make dormant cancer cells not properly safe and harmless. Dormancy allows cancer cells to evade the highly selective drug pressure as well as the immune response, to survive in an hostile environment and to awaken in more favorable conditions, leading to tumor or metastasis growth (8). Moreover, it has been intriguingly proposed that cancer dormancy is an intelligent adaptive behavior that prolongs the life of the host and then of the cancer itself (80). Quiescence could be the adaptive response of cancer cells to treatment with antitumor drugs, most of which target actively proliferating cells. For this reason, both non tumor and tumor cells in the tumor microenvironment and the (pre-)metastatic niche can release factors, including EVs, able to induce or reverse cancer cell dormancy, being both processes functional for cancer persistence and progression. Related to this, therapeutic approaches are also dualistic, involving the development of both sleeping and awakening strategies to reach the same goal, that is prevent tumor relapse and improve patient prognosis.

In this intriguing scenario, the identification of dormancy-associated molecules and related regulatory mechanisms would be crucial for both diagnostics and therapeutics. At the diagnostic level, these molecules could serve as reliable biomarkers for the specific detection of dormant cells before or at very early stages of re-awakening, while at the therapeutic level they could represent potential targets to be blocked in order to prevent tumor relapse. In both cases, approaches based on EV exploitation could be extremely useful. Since EVs are detectable in almost all body fluids, diagnostic methods based on liquid biopsy and sequential quantitative and qualitative analysis of EVs would be of great help in non-invasive monitoring of disease-free cancer patients. However, to move in this direction, current limitations in the standardization and reproducibility of analytical methods to identify and possibly sort EVs specifically associated with a particular feature or released by a particular cell subset need to be carefully addressed. From a therapeutic point of view, EVs could be extremely useful as drug delivery vectors in a targeted approach. To realize these strategies, more reliable and sustainable sources of EVs (not only MSCs, but also milk, plants and microalgae) have been identified, together with improvements in the associated techniques for EV isolation, engineering and delivery. Significant efforts have been made by the international EV community to standardize procedures and methodologies and to identify commonly agreed strategies at each level of the pipeline, from basic research to diagnostic and therapeutic applications (81–85) In addition, various EV-based biotechs and spin-offs have been established to exploit EV research (86). Ongoing work is likely to improve current knowledge of the contribution of EVs to dormancy/awakening fine-tuning, and to stimulate novel EV-based theranostic solutions, with the desirable goal of improving the prognosis and living conditions of cancer patients.
